# Respondent-Reported Acceptability and Tolerability of a Nurse-Assisted, Self-Collected Human Papillomavirus (HPV) Cervical Cancer Screening Model in Adults With Intellectual and Developmental Disabilities: A Preliminary Report

**DOI:** 10.7759/cureus.117558

**Published:** 2026-10-06

**Authors:** Zehra Rahman, Jusil Rice, Linda Edwards, Emma Sanad, Rafik Jacob

**Affiliations:** 1 Internal Medicine, University of Florida, Jacksonville, USA; 2 Biology, University of Florida, Gainsville, USA

**Keywords:** caregiver engagement, cervical cancer screening, health equity, human papillomavirus testing, intellectual and developmental disabilities, nursing workflow, preventive care access, self-collection

## Abstract

Background and objective

Adults with intellectual and developmental disabilities (IDD) are screened for cervical cancer at substantially lower rates than adults without disabilities. Communication differences, sensory sensitivities, and difficulty tolerating speculum-based examination all contribute. Deferred screening is frequently recorded as patient refusal rather than as an unmet need for adapted care. Human papillomavirus (HPV) testing on self-collected vaginal specimens is now recognized as an acceptable screening option, but published implementation strategies for this population are limited. The objective of this study was to evaluate respondent-reported acceptability, tolerability, and level of assistance required for a nurse-assisted self-collected HPV screening model in an interdisciplinary primary care clinic serving adults with IDD.

Methods

We conducted a prospective, single-site, minimal-risk observational study at the Program for Adults with Intellectual and Developmental Disabilities (PAIDD) at University of Florida Health Jacksonville. Vaginal specimens for HPV testing were collected in the clinic by the patient under nurse guidance, with hands-on assistance from a trained program nurse or a caregiver as needed. The model also included structured pre-visit planning, an animated patient education video, and caregiver engagement. A structured post-visit survey administered in REDCap assessed the level and appropriateness of assistance, patient tolerance, observable distress, acceptability, and support for future use. The survey was designed as a caregiver proxy instrument and was completed directly by the patient where able; outcomes are therefore described as respondent-reported, with proxy and direct responses distinguished explicitly. Results are reported as n (%). Recruitment is ongoing toward a planned enrollment of 30, and these are preliminary findings.

Results

Of 23 patients identified as eligible, 18 (78.3%) were offered the pathway. Among those offered, 14 (77.8%) completed collection, and four (22.2%) declined; zero (0%) attempted collection without completing it. Uptake was 14 of 23 (60.9%) among those identified as eligible. All 14 specimens (100%) returned negative high-risk HPV results. Complete survey data were available for 13 of the 14 completed collections. Among these 13 encounters, tolerance was rated very good in 11 (84.6%) and good in two (15.4%), with zero (0%) rated fair, poor, or very poor; no distress was observed in 12 (92.3%) and mild distress in one (7.7%), with zero (0%) moderate or severe. All 13 encounters with available assistance data (100%) required hands-on assistance, provided by the nurse in nine (69.2%) and by a caregiver in four (30.8%); zero (0%) were completed unaided or with verbal coaching alone, and zero (0%) were unable to be completed despite assistance. All 13 respondents (100%) judged the level of assistance appropriate, rated the process acceptable or very acceptable, and would support offering it again.

Conclusions

A nurse-assisted self-collected HPV screening model was well tolerated and highly acceptable to respondents in an academic primary care clinic for adults with IDD. Every encounter with available assistance data required hands-on assistance, indicating that nursing support is integral to the model rather than incidental. Clinic efficiency, physician time, and scalability were not measured and remain hypotheses for future evaluation. Recruitment continues.

## Introduction

Inclusive cervical cancer screening is a critical health equity issue. Adults with intellectual and developmental disabilities (IDD) are substantially under-screened for cervical cancer. A population-based study comparing women with and without IDD found significantly lower participation in cervical screening among women with IDD, leading the authors to question whether screening programs are delivered equitably to this population [1]. This disparity is multifactorial and reflects both patient-level and system-level barriers. Traditional speculum-based pelvic examination may be intolerable or distressing for some individuals with IDD. Deferred screening is then often mischaracterized as patient refusal rather than as a need for adapted care approaches. A prior review examined barriers to pelvic examination for cervical cancer screening in women with cerebral palsy, a group that overlaps substantially with the population served by this clinic [2].

Multiple patient-level and structural barriers contribute to this gap, including sensory sensitivities, communication differences, mobility limitations, anxiety related to pelvic examination, and limited availability of clinician time and training to support adapted screening workflows in primary care settings. A systematic review of cancer screening behavior among people with intellectual disabilities identified barriers and facilitators operating at the patient, caregiver, and health system levels, indicating that screening uptake in this population is shaped by modifiable factors rather than by patient preference alone [3].

In practice, options offered to patients who cannot tolerate speculum examination have included modified examination technique, sedation or anesthesia in selected cases, and greater caregiver involvement. Each is resource-intensive, and none is easily scalable in routine primary care.

Human papillomavirus (HPV) testing on a self-collected vaginal specimen offers an alternative pathway, and current guidance distinguishes it from clinician-collected primary HPV testing. The American Cancer Society recommends that individuals with a cervix begin cervical cancer screening at age 25 years and continue through age 65 years, with primary HPV testing on a clinician-collected cervical specimen every five years as the preferred strategy [4]. In a 2026 update, the American Cancer Society concluded that clinician-collected cervical specimens remain preferred, but that self-collected vaginal specimens are acceptable for cervical cancer screening in average-risk individuals aged 25-65 years, with repeat testing recommended at three years rather than five years following a negative self-collected result [5]. The World Health Organization recommends HPV DNA detection as the primary screening method for the general population of women and, as a conditional recommendation based on low-certainty evidence, suggests that HPV DNA testing may be performed on either a provider-collected or a self-collected sample [6]. A US Preventive Services Task Force draft recommendation statement addressing patient-collected high-risk HPV screening was posted for public comment on December 10, 2024; that draft has not been finalized and does not represent a current Task Force recommendation [7]. Published implementation experience describing how self-collected HPV screening can be operationalized for patients with significant sensory, cognitive, and behavioral barriers remains limited.

The aim of this work was to evaluate respondent-reported acceptability and tolerability of a clinic-based, nurse-assisted self-collected HPV screening model for patients with IDD within an academic primary care setting and to characterize the level of assistance required to complete collection.

Portions of this work were previously presented as a plenary presentation at the Society of General Internal Medicine Southern Regional Meeting (Inclusive Cervical Cancer Screening: Nurse-Led, Self-Administered Swab Model for Adults with Intellectual and Developmental Disabilities, 2026, New Orleans, LA, USA) and as a poster at the Down Syndrome Medical Interest Group-USA (DSMIG-USA) Annual Symposium (Implementing Nurse- and Caregiver-Supported Self-Collected HPV Screening in Adults with Intellectual and Developmental Disabilities, 2026, Orlando, FL, USA).

## Materials and methods

Study design and setting

This was a prospective, single-site, minimal-risk observational study evaluating the feasibility and acceptability of an adapted cervical cancer screening workflow offered as part of routine clinical care. It was conducted at the Program for Adults with Intellectual and Developmental Disabilities (PAIDD) at University of Florida Health Jacksonville, an interdisciplinary primary care medical home led by a physician medical director, operationalized by a registered nurse (RN) program coordinator, and delivered by advanced practice providers, physicians, and nursing staff in close partnership with caregivers. The program was established in 2013 and serves approximately 350 adults with IDD, many of whom are nonverbal or minimally verbal and have significant sensory sensitivities and anxiety related to clinical examination. Approximately 40% of the program population are women, and approximately 80% of those fall within the 25- to 64-year screening age range, giving an estimated 110 potentially eligible patients. These are program-level estimates rather than counts from a verified screening registry. Data were collected between February 3, 2026 and August 25, 2026. Recruitment is ongoing.

Participants and eligibility

Patients were eligible if they were adults aged 25-64 years receiving care at the PAIDD clinic, had a diagnosis of intellectual and/or developmental disability, had a cervix, were due for cervical cancer screening per clinic practice, and if a caregiver was available and willing to complete a proxy survey. The age range was chosen to align with American Cancer Society guidance recommending initiation of screening at age 25 years [4,5]. Screening was deferred where there was a medical contraindication to HPV testing, where the clinical team determined that screening was not appropriate that day, or where the patient exhibited behavioral distress such that proceeding was not clinically appropriate. Patients for whom an HPV-only pathway is not appropriate under current guidance, including those with HIV infection or another immunocompromising condition, a history of high-grade cervical dysplasia, or in utero diethylstilbestrol (DES) exposure, would have been managed with alternative screening strategies; 0 patients (0%) in the screened population met any of these criteria.

Intervention

The model was designed to address the barriers most commonly encountered in patients with IDD: difficulty tolerating speculum-based examination, communication differences, and reliance on caregivers for coordination and decision-making. HPV-based screening was prioritized because it identifies high-risk HPV infection before cytologic change and, on a self-collected vaginal specimen, avoids speculum insertion entirely. Patients screened through this pathway are advised to repeat testing at three years following a negative result, consistent with the interval recommended for self-collected vaginal specimens, rather than the five-year interval that applies to clinician-collected primary HPV testing [5]. In the HPV FOCAL randomized trial, primary HPV testing on clinician-collected specimens resulted in a significantly lower likelihood of cervical intraepithelial neoplasia grade 3 or higher at 48 months than liquid-based cytology; invasive cervical cancer incidence was not an endpoint of that trial [8]. A meta-analysis of HPV testing on self-collected compared with clinician-collected samples found lower pooled sensitivity and specificity on self-collected samples overall, with the difference concentrated in signal amplification assays; the authors concluded that some polymerase chain reaction (PCR)-based assays could be considered for routine screening after careful piloting [9]. The assay used in this study is PCR-based.

The model had four components: (1) adoption of HPV screening on a self-collected vaginal specimen to reduce procedural burden; (2) structured pre-visit planning to identify eligible patients and anticipate support needs; (3) patient self-collection of the specimen as the default, with expansion of nursing roles to include patient preparation, positioning assistance, real-time coaching, and assisted collection when needed; and (4) integration of caregivers to support communication, facilitate the consent and assent process, and enhance patient comfort.

Implementation proceeded through a multidisciplinary process. Attending physicians, resident physicians, nursing staff, and caregivers were engaged early to identify workflow barriers and inform intervention design. Clinic workflows were modified to include pre-visit identification of patients due for screening and to allocate additional appointment time for encounters requiring enhanced support. Nursing staff received targeted training on HPV screening workflows and on strategies for supporting patients with IDD, including communication techniques and assistance during specimen collection. Roles and responsibilities were delineated in advance to promote consistency across providers. Figure 1 illustrates the adapted clinic workflow.

**Figure 1:**
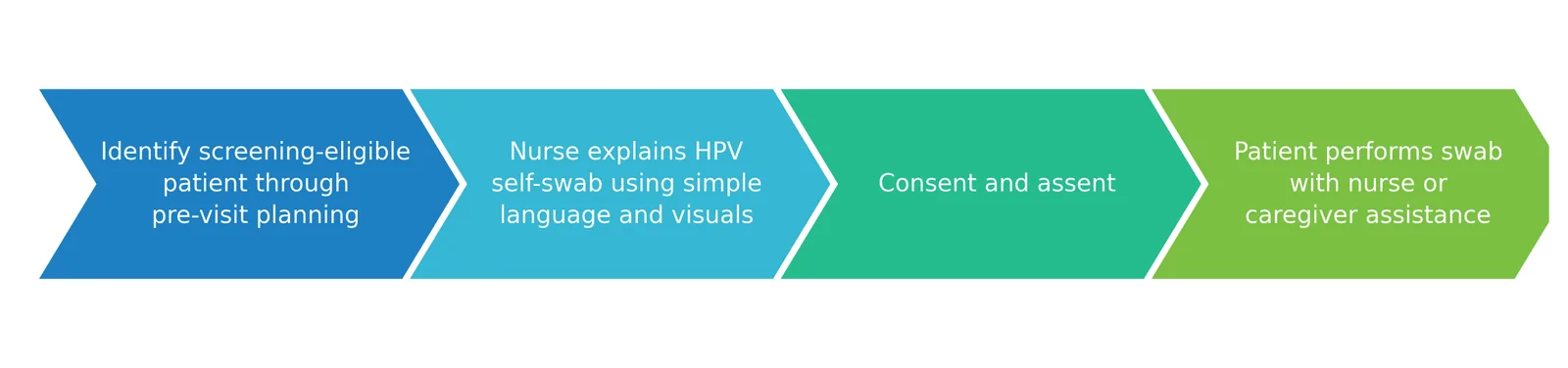
Adapted clinic workflow for nurse-assisted self-collected HPV screening. Adapted clinic workflow incorporating pre-visit identification of screening-eligible patients, nurse-delivered education using accessible language and visuals, structured consent and assent, and specimen collection performed by the patient with hands-on assistance from the program nurse or a caregiver.

Specimen collection

Vaginal specimens were collected using the OneSwab collection kit (Medical Diagnostic Laboratories, Hamilton, NJ, USA), a snap-swab device with a screw-cap design permitting room-temperature storage and transport. Collection took place in the clinic examination room rather than at home, so that support was available at the point of collection.

Specimens were processed by Medical Diagnostic Laboratories, a CLIA-certified reference laboratory, using the HPV Type-Detect 4.0 multiplex real-time PCR assay. The assay identifies and simultaneously distinguishes 13 high-risk HPV genotypes: 16, 18, 31, 33, 35, 39, 45, 51, 52, 56, 58, 59, and 68. This assay is a laboratory-developed test; its performance characteristics were determined by the laboratory, and it has not been cleared or approved by the U.S. FDA.

A graded approach to collection was used, with support matched to the individual patient rather than withheld until failure. Collection was performed by the patient under nurse guidance, in privacy, and with verbal coaching. The PAIDD program nurse, trained in the protocol and in supporting patients with IDD, provided hands-on assistance or completed the collection where the patient could not do so independently, which was anticipated for a substantial proportion of this population. Caregiver assistance was used where the patient preferred it or where caregiver presence was necessary for the patient to proceed. Nurse assistance was preferred over caregiver assistance where either was acceptable to the patient, as the nurse is trained in specimen adequacy and in de-escalation. No patient was required to undergo speculum insertion to complete screening. Because hands-on assistance was required in every encounter for which assistance data were available, the specimen is described throughout as nurse-assisted self-collected rather than as unassisted self-collected.

Patient education materials

Patient preparation included an animated education video explaining the self-collection procedure in accessible, trauma-informed language. The video uses an animated character rather than clinical photography or live demonstration, conveying the steps of the procedure without exposure to imagery that patients with sensory sensitivities may find distressing. It was developed from an original concept and storyboard by the senior author (RF) and produced with University of Florida Health Jacksonville Media Relations. The video was shown to patients and caregivers before the encounter and was available for repeat viewing at home. Video 1 presents the education video in full.

**Video 1:** Patient and caregiver education video for nurse-assisted self-collected HPV cervical cancer screening. Animated education video shown to patients and caregivers before the screening encounter, explaining the self-collection procedure in accessible, trauma-informed language. The video uses an animated character rather than clinical photography or live demonstration, conveying the steps of the procedure without exposure to imagery that patients with sensory sensitivities may find distressing. HPV, human papillomavirus Developed from an original concept and storyboard by the senior author (RF) and produced with University of Florida Health Jacksonville Media Relations.

Data sources

Two data sources were used. Respondent-reported outcomes were obtained from the post-visit survey described below. Counts of patients identified as eligible, offered the pathway, completing collection, and declining were compiled from the clinic’s pre-visit planning records maintained as part of routine clinical operations and are reported here in aggregate without identifiers.

Survey instrument and data collection

A structured post-visit survey was completed at the end of the encounter. The instrument assessed the level of assistance required from nursing staff and from caregivers, whether the level of assistance provided was appropriate, overall tolerance of the self-swab process, observable signs of distress, overall acceptability of the method, and willingness to use the approach again. The instrument was investigator-developed and was not formally validated. It was administered by the program nurse through an anonymous REDCap link immediately following the encounter, with REDCap being a secure, institutionally hosted electronic data capture platform. Study data were collected and managed using REDCap electronic data capture tools hosted at the University of Florida [10,11]. The full instrument is presented in Appendix A.

The instrument was designed as a caregiver proxy survey, and caregivers responded as proxies for most encounters, reporting their own observations rather than the patient’s direct report, as many patients in this population are nonverbal or minimally verbal. Where the patient was able to respond, the patient completed the survey directly rather than through a proxy. Because the dataset therefore combines proxy report and direct patient report, outcomes are described throughout as respondent-reported rather than as caregiver-reported, and the distribution of respondent roles, including the proportion responding directly, is given in the Results.

In REDCap, a survey record is created at the moment a survey link is generated rather than at the moment a response is submitted. Links were generated at the time of pre-visit planning for encounters at which screening was anticipated, before eligibility and the offer of the pathway were confirmed at the visit, and a further link was generated where a patient returned at a later visit. The number of records generated therefore exceeds the number of patients identified as eligible and the number offered the pathway, and the records correspond to survey link generations rather than to unique patients.

Consent was documented separately in the patient’s medical record; survey responses recorded in REDCap contained no names, medical record numbers, or other identifiers and were not linked to the consent documentation. All caregivers present at an eligible screening encounter during the evaluation period were invited to complete the survey.

Statistical analysis

One record submitted during instrument testing, containing non-substantive free-text entries and predating the first patient encounter, was excluded before analysis. Survey links generated but never opened were treated as non-responses. Responses were summarized using descriptive statistics and are reported throughout as n (%), with the denominator stated for each domain; missing responses were not imputed. Because the survey dataset is anonymous and cannot be linked to the clinical record, outcomes for the one completed collection without complete survey data could not be recovered from another source, and assistance, tolerance, distress, and acceptability results are therefore reported against the 13 encounters with available data rather than against all 14 completed collections. Given the pilot design and small sample, no inferential testing was performed, and no comparison group was used. A subset of these data was presented previously in preliminary form; the proportions reported here differ from those presentations because the test record described above was excluded from the present analysis.

Clinic-level cervical cancer screening completion rates before and after implementation are being collected in parallel and will be reported separately. The study has a planned enrollment of 30 participants. Recruitment had not closed at the time of this report, and the results presented here are preliminary, reflecting the first 14 completed screenings and the survey responses returned for them.

Ethical considerations

This project was reviewed by the University of Florida Institutional Review Board and determined to be exempt under 45 CFR 46.104(d)(2), which covers research involving survey procedures where responses are recorded without identifiers (IRB202501845). The materials submitted to the Institutional Review Board comprised the study protocol, the consent documentation, and the caregiver proxy survey instrument presented in Appendix A.

Consent for participation was obtained from the patient where the patient was capable of providing it, and otherwise from a power of attorney, legally authorized representative, or healthcare surrogate. Consent was documented separately in the patient’s medical record and was not linked to the de-identified research dataset. Patient assent was sought in all cases, including for nonverbal patients through observable acceptance or refusal cues, and collection was paused or stopped if the patient showed escalating distress or indicated refusal. Because specimen collection itself takes only a few seconds, an encounter in which observable distress was recorded was in some cases already complete at the point the distress was noted; observed distress and collection completion were therefore recorded as separate measures rather than as a single outcome. Research consent was obtained by the investigators separately from routine clinical care discussions; participation was voluntary and did not affect clinical care, and no incentives were offered.

## Results

Screening completion

These are preliminary results from an ongoing study. Twenty-three patients were identified as eligible for cervical cancer screening through pre-visit planning during the reporting period. Of these, 18 (78.3%) were offered the nurse-assisted self-collection pathway. Among those offered, 14 (77.8%) completed collection and four (22.2%) declined; zero (0%) attempted collection without completing it, and zero (0%) were unable to complete collection despite assistance. Uptake was 14 of 18 (77.8%) among those offered and 14 of 23 (60.9%) among those identified as eligible. The screening cascade is presented in Table 1.

**Table 1:** Screening cascade for nurse-assisted self-collected HPV screening. Number of patients at each stage of the screening pathway during the reporting period, compiled from clinic pre-visit planning records. Percentages are shown relative to the 23 patients identified as eligible and to the 18 patients offered the pathway. Dashes indicate that the denominator does not apply. HPV, human papillomavirus

Stage	n	% of those identified (n = 23)	% of those offered (n = 18)
Identified as eligible through pre-visit planning	23	100	-
Offered the nurse-assisted self-collection pathway	18	78.3	100
Completed specimen collection	14	60.9	77.8
Declined participation	4	17.4	22.2
Attempted collection but did not complete	0	0	0
Unable to complete despite assistance	0	0	0
Specimen reported inadequate by the laboratory	0	0	0
High-risk HPV detected (of 14 completed collections)	0	-	-

All 14 specimens (100%) returned negative high-risk HPV results. No specimen (0%) was reported by the laboratory as inadequate or unsatisfactory for testing, and no patient (0%) required referral for colposcopy or speculum-based evaluation.

Survey response

Twenty-seven survey records were generated in REDCap. As described in the Methods, a record is created when a survey link is generated rather than when a response is submitted, and links were generated at pre-visit planning before eligibility, and the offer was confirmed at the visit, so the number of records exceeds the number of patients identified as eligible and the number offered the pathway. Of the 27 records, 11 (40.7%) links were never opened, and one (3.7%) was a test submission excluded before analysis, leaving 15 (55.6%) records with data: 14 corresponding to completed collections and one documenting an encounter at which collection was not attempted. Complete experience data were available for 13 of the 14 completed collections (92.9%); one respondent (7.1%) recorded the encounter but did not submit the remaining items. Denominators are reported accordingly, and missing responses were not imputed.

Among the 15 records with data, respondents were family members in four (26.7%), direct support professionals in four (26.7%), legal guardians in two (13.3%), and others in five (33.3%). Of those recorded as other, three were patients responding about their own experience. Caregivers or other proxies were therefore the respondents in 12 of the 15 records with data (80.0%), and the patient responded directly in three (20.0%). Because the dataset combines proxy report and direct patient report, outcomes are described as respondent-reported, and the two response sources are distinguished wherever they are reported.

Respondent-reported outcomes

Respondent-reported outcomes for the 13 encounters with complete survey data are presented in Table 2.

**Table 2:** Respondent-reported outcomes of nurse-assisted self-collected HPV screening. Responses to the structured post-visit survey for the 13 encounters with complete survey data, reported as n (%). Assistance, tolerance, distress, and acceptability data were not available for the fourteenth completed collection. Response options that received no responses are shown as 0 (0) so that the full range of each item is visible. Respondents were caregivers or other proxies in 12 of the 15 records with data (80.0%) and the patient directly in three (20.0%). The survey instrument is reproduced in Appendix A. HPV, human papillomavirus

Domain	Response	n (%)
Overall tolerance of the self-swab process	Very good	11 (84.6)
	Good	2 (15.4)
	Fair	0 (0)
	Poor	0 (0)
	Very poor	0 (0)
Observable signs of distress	None observed	12 (92.3)
	Mild	1 (7.7)
	Moderate	0 (0)
	Severe (procedure stopped or nearly stopped)	0 (0)
Level of assistance required	No assistance	0 (0)
	Verbal coaching only	0 (0)
	Hands-on assistance from caregiver	4 (30.8)
	Hands-on assistance from nurse	9 (69.2)
	Unable to complete despite assistance	0 (0)
Level of assistance judged appropriate	Yes	13 (100)
	No	0 (0)
	Not sure	0 (0)
Overall acceptability of the method	Very acceptable	8 (61.5)
	Acceptable	5 (38.5)
	Neutral	0 (0)
	Unacceptable	0 (0)
	Very unacceptable	0 (0)
Would support offering the method again	Yes	13 (100)
	No	0 (0)
	Not sure	0 (0)

Overall tolerance of the self-swab process was rated very good in 11 (84.6%) and good in two (15.4%); zero (0%) were rated fair, poor, or very poor. No observable signs of distress were reported in 12 (92.3%), and mild distress in one (7.7%), manifesting as agitation; zero (0%) were rated moderate or severe distress, and in no case was the procedure stopped or nearly stopped because of distress.

All 13 encounters with available assistance data (100%) required hands-on assistance to complete collection, provided by the program nurse in nine (69.2%) and by a caregiver in four (30.8%). No encounter (0%) was completed with verbal coaching alone or without assistance, and zero (0%) were unable to be completed despite assistance. Assistance data were not available for the fourteenth completed collection, and because the survey dataset is anonymous, these outcomes could not be recovered from the clinical record. All 13 respondents (100%) judged the level of assistance provided to be appropriate for the needs of the patient.

All 13 respondents (100%) rated the process as acceptable or very acceptable, with eight (61.5%) rating it very acceptable and five (38.5%) acceptable; zero (0%) rated it neutral, unacceptable, or very unacceptable. All 13 (100%) indicated they would support this screening method being offered again in the future. One respondent noted appreciation that an alternative to Papanicolaou testing was available.

Because these proportions derive from 13 responses, a single response shifts a proportion by approximately 7.7 percentage points, and the findings describe this pilot cohort rather than providing precise estimates.

## Discussion

This work demonstrates that a nurse-assisted workflow can be used to deliver HPV cervical cancer screening on a self-collected vaginal specimen to adults with IDD in routine primary care. Self-collected HPV screening is now recognized in national guidance as an acceptable option for average-risk individuals, but its implementation in populations with significant sensory, cognitive, and behavioral barriers remains limited. This report describes a pragmatic implementation strategy rather than a novel diagnostic or technological intervention.

A key feature of the approach is the integration of nursing staff into the screening workflow to support patient preparation and specimen collection. Caregiver involvement supports communication, patient comfort, and completion of screening within a single encounter, which is relevant in a population for whom additional visits carry transportation, scheduling, and anxiety burdens.

Among patients offered the pathway, 14 of 18 (77.8%) completed collection, no attempt that began was left unfinished, and no patient proved unable to complete collection despite assistance. Four patients (22.2% of those offered) declined. Declination is reported here as a legitimate outcome of a model built on assent rather than as a failure of the workflow. These proportions describe completion among patients offered a specific pathway at a clinic encounter and are not comparable with population-level cervical cancer screening coverage estimates, which use different denominators, timeframes, and ascertainment methods; no such comparison is drawn here.

The most consequential finding is that no encounter with available assistance data was completed unaided or with verbal coaching alone. All 13 such encounters required hands-on assistance, from the nurse in nine (69.2%) and from a caregiver in four (30.8%). Self-collection in this population is therefore better understood as nurse-assisted collection: the term describes who holds the swab less than it describes a procedure that removes the speculum, the stirrups, and the clinician-directed examination while retaining hands-on support. This distinction matters for anyone planning to replicate the model, because a clinic that budgets for unassisted self-collection will not be able to deliver it. It also means that the accuracy literature on self-collected specimens [9] bears on the specimen type used here rather than on the staffing model.

Acceptability was uniform among respondents. Every respondent rated the process acceptable or very acceptable, judged the assistance provided appropriate, and would support offering it again, and tolerance was rated good or very good in every encounter with survey data. Observable distress was rare and mild where present, with no encounter requiring the procedure to be stopped. In a population where speculum examination is frequently deferred or performed under sedation, a screening pathway that no respondent found unacceptable is a substantive result even in a small cohort.

The model requires no additional equipment or capital investment beyond the collection kit and aligns with existing preventive care reimbursement structures, which suggests it may be transferable to other primary care settings, including academic clinics, federally qualified health centers, and community practices. Clinic efficiency, physician time, staffing cost, and screening uptake at the clinic level were not measured in this study; however, the scalability of the model therefore remains a hypothesis rather than a finding. The requirement for hands-on nursing assistance in every encounter with available data is itself a resource implication that any replicating site would need to plan for.

The animated education video is a low-cost component that is independently transferable. Accessible preparation materials address a barrier that procedural adaptation alone does not, since informed assent requires that the patient understand what the procedure involves. The video is publicly available and can be adopted by other clinics without cost, although its independent contribution was not evaluated in this study.

The approach supports more inclusive, sensory-informed care for patients who may otherwise be unable to tolerate or complete speculum-based examination. It emphasizes structured assent, caregiver partnership, and minimization of distress, consistent with principles of patient-centered and disability-affirming care.

Limitations

This evaluation has several limitations. It was conducted at a single academic site with an established interdisciplinary IDD program, and the findings may not generalize to settings without comparable nursing capacity or caregiver engagement. The sample was small, with 14 completed collections against a planned enrollment of 30, and surveys were completed at the end of the encounter, which may favor positive reporting. One respondent recorded the encounter but did not submit the experience items, so those outcomes describe 13 of the 14 completed collections (92.9%); because the survey dataset is anonymous by design, the missing outcomes could not be recovered from the clinical record, and that encounter may have differed from those reported. The uniformly positive ratings should also be read with the setting in mind: respondents were reporting on a service delivered by a team they know, in a program they have chosen to remain with, which is a context that discourages criticism. The survey instrument was investigator-developed and was not formally validated or pilot-tested, so its measurement properties are unknown. Experience was reported largely by caregivers acting as proxies rather than by patients directly, a limitation in a population whose own preferences are underrepresented in the literature, although three respondents were patients reporting on their own experience. There was no comparison group and no pre-intervention acceptability measure, so the results describe acceptability of the adapted approach rather than improvement relative to usual care. Clinic-level screening uptake data were still being collected at the time of this report, so effectiveness cannot yet be assessed. No high-risk HPV infections were detected in this cohort, so the downstream pathway for managing an abnormal result, including speculum-based evaluation or anesthesia-assisted follow-up, was not tested in practice.

The HPV assay used here is a laboratory-developed test rather than an assay cleared by the FDA for primary cervical cancer screening. It detects 13 high-risk genotypes and does not include HPV 66, which is covered by some FDA-approved primary screening assays. Pooled accuracy of HPV testing on self-collected specimens is lower than on clinician-collected specimens overall, with the difference concentrated in signal amplification rather than PCR-based assays [9]. The assay used here is PCR-based, but clinics replicating this model should confirm that their laboratory has validated its assay for self-collected vaginal specimens. Clinically, the approach does not eliminate the need for speculum-based evaluation when HPV results are abnormal, and some patients may still require anesthesia-assisted procedures for follow-up. Because the recommended repeat interval following a negative self-collected result is three years rather than five, the reduction in lifetime procedural burden relative to clinician-collected primary HPV testing is smaller than the avoidance of a single speculum examination might suggest [5]. Individuals with HIV infection or immunocompromising conditions may have impaired viral clearance and are not ideal candidates for HPV-only screening pathways. Patients with prior high-grade cervical disease or in utero DES exposure likewise require alternative screening strategies.

## Conclusions

Cervical cancer screening disparities among adults with IDD are driven substantially by modifiable system-level barriers rather than by patient refusal. A nurse-assisted model of self-collected HPV screening was feasible within an existing interdisciplinary primary care clinic and was well received: 14 of 18 patients offered the pathway (77.8%) completed collection, tolerance was rated good or very good in all 13 encounters (100%) with complete survey data, observable distress was rare and mild, and all 13 respondents (100%) judged the process acceptable and would support offering it again. Every encounter with available assistance data required hands-on assistance, which should be planned for rather than treated as an exception, and the model is therefore more accurately described as nurse-assisted self-collection. Whether the model improves clinic efficiency, preserves physician time, or is scalable beyond this setting was not measured and remains to be tested. Recruitment toward the planned enrollment of 30 is continuing, and final results will be reported on completion. Further work should evaluate screening uptake before and after implementation, downstream clinical outcomes, patient-reported rather than proxy-reported experience, and sustainability across diverse care settings.

## Appendices

Appendix A

**Table 3:** Post-visit survey instrument. Investigator-developed 10-item instrument administered through an anonymous REDCap link immediately following the screening encounter. The instrument was designed as a caregiver proxy survey and was completed directly by the patient where the patient was able to respond. It was not formally validated or pilot-tested.

Item	Question	Response options
1	What is your role in relation to the patient?	Family member; Legal guardian; Direct support professional; Other (please specify)
2	Was the HPV self-swab attempted during today’s visit?	Yes, completed; Yes, attempted but not completed; No, not attempted
3	How would you rate the patient’s overall tolerance of the self-swab process?	Very good; Good; Fair; Poor; Very poor
4	Did the patient show signs of distress during the process?	No distress observed; Mild distress; Moderate distress; Severe distress (procedure stopped or nearly stopped)
5	Which signs of distress were observed? (select all that apply)	Verbal refusal; Physical resistance; Anxiety or fear; Agitation; Other (please specify)
6	What level of assistance did the patient require?	No assistance; Verbal coaching only; Hands-on assistance from caregiver; Hands-on assistance from nurse; Unable to complete despite assistance
7	In your opinion, was the level of assistance appropriate for the patient’s needs?	Yes; No; Not sure
8	Overall, how acceptable was the HPV self-swab process for the patient?	Very acceptable; Acceptable; Neutral; Unacceptable; Very unacceptable
9	Would you support this screening method being offered again in the future?	Yes; No; Not sure
10	Please share any additional comments or observations about the patient’s experience (optional)	Free text

## References

[1] Cobigo V, Ouellette-Kuntz H, Balogh R, Leung F, Lin E, Lunsky Y (2013). Are cervical and breast cancer screening programmes equitable? The case of women with intellectual and developmental disabilities. J Intellect Disabil Res.

[2] Fasen M, Saldivar B, Elamsenthil S, Thompson J, Fouad L, Edwards L, Jacob R (2020). Gynecological care and contraception considerations in women with cerebral palsy. South Med J.

[3] Chan DN, Law BM, Au DW, So WK, Fan N (2022). A systematic review of the barriers and facilitators influencing the cancer screening behaviour among people with intellectual disabilities. Cancer Epidemiol.

[4] Fontham ET, Wolf AM, Church TR (2020). Cervical cancer screening for individuals at average risk: 2020 guideline update from the American Cancer Society. CA Cancer J Clin.

[5] Perkins RB, Wolf AM, Church TR (2026). Self-collected vaginal specimens for human papillomavirus testing and guidance on screening exit: an update to the American Cancer Society cervical cancer screening guideline. CA Cancer J Clin.

[6] (2026). WHO guideline for screening and treatment of cervical pre-cancer lesions for cervical cancer prevention. https://www.who.int/publications/i/item/9789240030824.

[7] (2026). Cervical cancer: screening. https://www.uspreventiveservicestaskforce.org/uspstf/draft-recommendation/cervical-cancer-screening-adults-adolescents.

[8] Ogilvie GS, van Niekerk D, Krajden M (2018). Effect of screening with primary cervical HPV testing vs cytology testing on high-grade cervical intraepithelial neoplasia at 48 months: the HPV FOCAL randomized clinical trial. JAMA.

[9] Arbyn M, Verdoodt F, Snijders PJ (2014). Accuracy of human papillomavirus testing on self-collected versus clinician-collected samples: a meta-analysis. Lancet Oncol.

[10] Harris PA, Taylor R, Thielke R, Payne J, Gonzalez N, Conde JG (2009). Research electronic data capture (REDCap)—a metadata-driven methodology and workflow process for providing translational research informatics support. J Biomed Inform.

[11] Harris PA, Taylor R, Minor BL (2019). The REDCap consortium: building an international community of software platform partners. J Biomed Inform.

